# Is the course of the cervicofacial division of the facial nerve consistent with the adjacent vessels?

**DOI:** 10.1002/ccr3.6952

**Published:** 2023-02-10

**Authors:** Alexandros Poutoglidis, George K. Paraskevas, Nikolaos Anastasopoulos, Irene Asouhidou, Paraskevi Karamitsou, Evropi Forozidou, Nikolaos Lazaridis

**Affiliations:** ^1^ Department of Otorhinolaryngology – Head and Neck Surgery ‘G. Papanikolaou’ General Hospital Thessaloniki Greece; ^2^ Department of Anatomy and Surgical Anatomy, Faculty of Health Sciences, Medical School Aristotle University of Thessaloniki Thessaloniki Greece

**Keywords:** aberrancy, facial nerve, neck dissection, parotid surgery

## Abstract

Individual facial nerve branching patterns can be difficult to predict. The superficial course of its terminal branches poses them at risk of injury during head and neck surgeries. We report the rare course of a branch of the facial nerve deep into the posterior facial vein.

## Graphical abstract

The facial nerve is well known for its aberrant course and wide variability. The surgeon should be aware of the variability of the relationship between the posterior facial vein and the mandibular branch of the facial nerve during dissection.

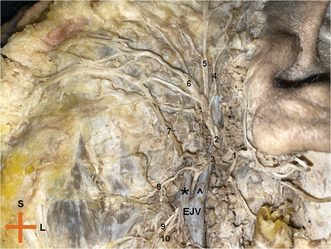

## 1 CASE PRESENTATION

**FIGURE 1:**
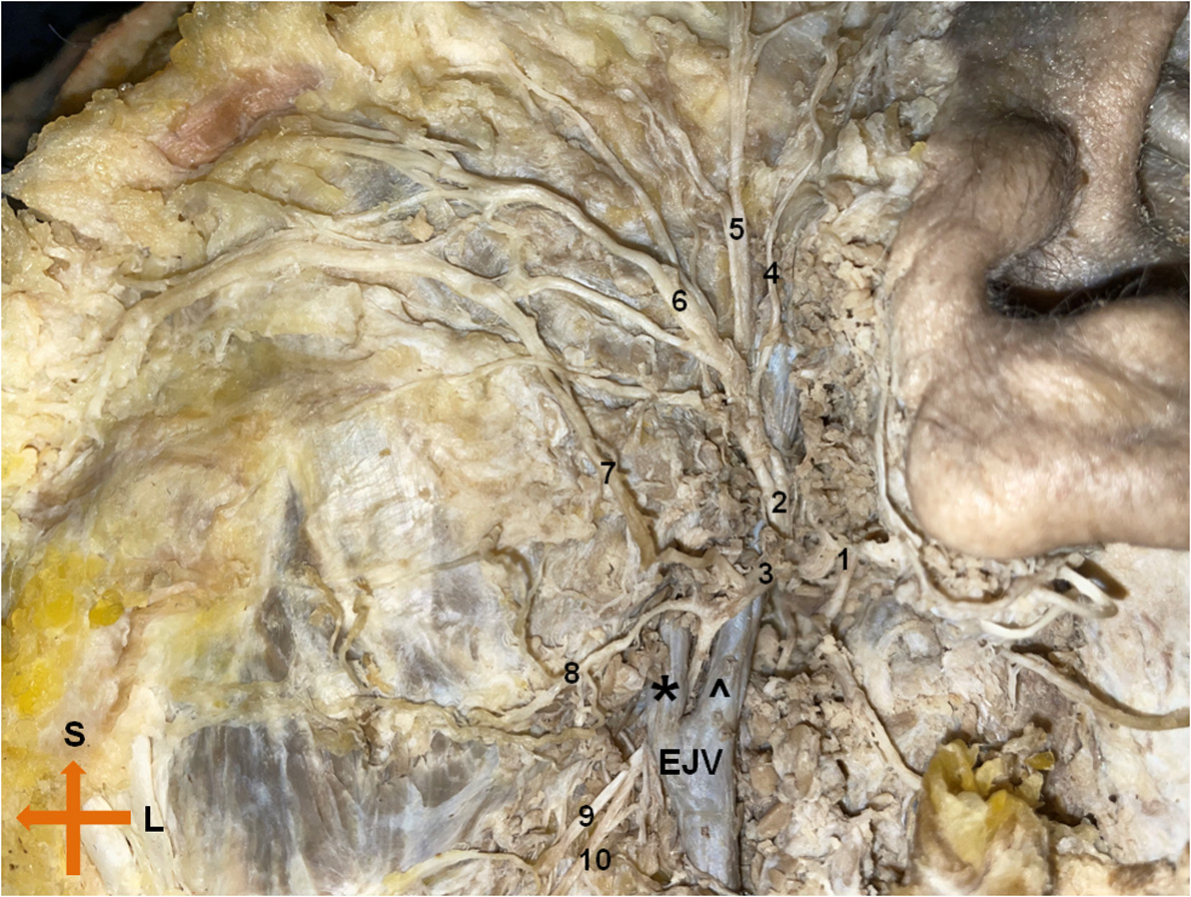
The left side of the extratemporal segment of facial nerve. 1: Main trunk of facial nerve, 2: Temporofacial division of facial nerve, 3: Cervicofacial division of facial nerve, 4: Temporal branch, 5: Zygomatic branch, 6: Buccal branch, 7: Anastomotic branch of cervicofacial division to the buccal branch, 8: Upper marginal branch, 9: Lower marginal branch, 10: Cervical branch. *, Posterior facial vein; ^, Retromandibular vein; EJV, external jugular vein; L, Lateral; S, Superior.

A rare course of the inferior division of the extratemporal facial nerve was observed during the dissection of a 75‐year‐old Caucasian female formalin embalmed Greek cadaver, donated to the Department of Anatomy, Aristotle University of Thessaloniki for teaching and research purposes. Heart attack was reported as the cause of death. No gross pathology or tumor formation was observed during the routine dissection of the head and neck region. The full dissection of the extratemporal portion of the facial nerve—after removal of the superficial lobe of the parotid gland—was the objective of the dissection, in an effort to map facial nerve branching patterns in the Greek population. The branching pattern of the facial nerve was type VI bilaterally according to Davis classification. On the left side, the inferior division of the main trunk (cervicofacial segment) was superficial to the retromandibular vein as usual. After giving an anastomotic branch to the buccal branch of the facial nerve a bifurcation was noted. The upper branch ran superficial to the posterior facial vein providing nerve supply to the corresponding angular oris muscle. The lower branch ran deep to the posterior facial vein and in the further 0.2 cm, it was divided into an upper marginal and a lower cervical branch (Figure 1). External jugular vein was coursed underneath the above bifurcation of the lower branch of facial nerve. Every branch of the facial nerve crossed horizontally beneath the investing layer of the deep cervical fascia and above the masseter muscle. No other anatomic variations were recorded. To the best of our knowledge, this is a very rare course of the inferior division of the facial nerve. All physicians and especially surgeons involved in the treatment of this area should be aware of all possible variations in order to ensure a safe diagnosis and an uneventful procedure in parotid or facial plastic surgery cases.

## 2 DISCUSSION

The extratemporal portion of the facial nerve presents a wide anatomic variability with anastomotic branches and various branching patterns. Davis classification still remains the most popular one, although many researchers did suggest other classifications.^1^ The analysis of anatomical variations contributes to obtaining an actual image of the human body inside, which is crucial in everyday clinical practice.^2^ Deep knowledge of the head and neck anatomy is a prerequisite in order to proceed to an uneventful and safe parotid surgery. Injury to the facial nerve leads to temporary or permanent palsy, with both functional and cosmetic sequelae. A safe superficial parotidectomy is performed after full facial neck dissection and excision of the tissue overlying the nerve.^3^ The retromandibular vein is the most consistent radiological landmark between the superficial and deep lobe of the parotid gland. Facial nerve superficial to the retromandibular vein and to the posterior facial vein. Thus, an anatomical variation with the nerve running beneath the posterior facial vein may lead to a risky dissection during parotid surgery and to an inadvertent injury after ligation of the external jugular vein. The presented variation highlights and augments the necessity of a meticulous dissection taking into account the highly possible presence of an underlying aberrancy.

## AUTHOR CONTRIBUTIONS

**Alexandros Poutoglidis:** Conceptualization; data curation; formal analysis; investigation; methodology; supervision; validation; visualization; writing – original draft; writing – review and editing. **George K. Paraskevas:** Conceptualization; data curation; validation; visualization; writing – original draft. **Nikolaos Anastasopoulos:** Conceptualization; data curation; formal analysis; investigation; methodology; validation; writing – review and editing. **Irene Asouhidou:** Conceptualization; data curation; validation; writing – review and editing. **Paraskevi Karamitsou:** Conceptualization; data curation; investigation; methodology; writing – original draft. **Evropi Forozidou:** Conceptualization; data curation; investigation; methodology; validation; writing – review and editing. **Nikolaos Lazaridis:** Conceptualization; data curation; investigation; methodology; supervision; validation; visualization; writing – original draft; writing – review and editing.

## FUNDING INFORMATION

None.

## CONFLICT OF INTEREST STATEMENT

No potential conflict of interest was reported by the authors.

## ETHICS STATEMENT

This is a cadaveric study. The cadaver was donated to our department for scientific and research purposes in a cadaver donation program, after the written consent of the relatives of the cadaver.

## CONSENT

Written informed consent was obtained from the patient to publish this report in accordance with the journal's patient consent policy.

## Data Availability

Data are available from the corresponding author with the permission of Aristotle University of Thessaloniki. The data that support the findings of this study are available from the corresponding author upon reasonable request.
